# Association between Body Weight and Body Mass Index and Patellar Tendinopathy in Elite Basketball and Volleyball Players, a Systematic Review and Meta-Analysis

**DOI:** 10.3390/healthcare10101928

**Published:** 2022-09-30

**Authors:** Minghao Deng, Michael Mansfield

**Affiliations:** School of Sports, Exercise and Rehabilitation Sciences, College of Life and Environmental Sciences, University of Birmingham, Birmingham B15 2TT, UK

**Keywords:** Body Weight (BW), Body Mass Index (BMI), Patellar Tendiopathy (PT), elite basketball players, elite volleyball players

## Abstract

The features of Patellar-Tendinopathy are (1): pain localised to the inferior pole of the patellar; (2): the presence of load-related pain. Body-Weight and Body-Mass-Index, as two easily-measured variables, could potentially aid the prediction of PT. This review aims to establish relationships between Body-Weight and Body-Mass-Index and Patellar-Tendinopathy via synthesising the evidence from prospective-cohort and cross-sectional studies in elite basketball and volleyball players. Seven databases (PubMed, EMBASE, CINAHL, Google Scholar, Health-Management-Information-Consortium, National-Technical-Information-Service, ClinicalTrial.gov) and citation chasing were used to identify English peer-review articles from 2000 to 2022. An adapted version of the Newcastle-Ottawa scale was used for critical appraisal. Two reviewers were involved in literature searching, data extraction, and quality review. Two prospective cohort and five cross-sectional studies met the inclusion criteria, providing 849 subjects (male:female: 436:413). Five studies found BW is associated with PT. Three studies found a relationship between BMI and PT. Six out of seven studies were classified as very good studies. All studies were level IV evidence. The very low certainty evidence suggests an association between BW and PT. There is moderate certainty evidence that BMI is associated with PT. These preliminary findings should be treated cautiously due to the lack of strong evidence.

## 1. Introduction 

Patellar tendinopathy (PT) is typically referred to as weakened and thickened patellar tendon due to the incomplete tendon healing after overuse injury [1]. It is also the common cause of activity-related anterior knee pain [2], unlike the patellar tendinitis in which the substance of the tendon exhibits a primary inflammation response [3]. The clinical features of PT are (1): pain localised to the inferior pole of the patellar [4]; (2): the presence of load-related pain with the demand of knee extensors, especially in the activities like jumping that requires the storage and release of energy of tendon [5]. The pathology change in the tendon can be defined as a failure in the homeostatic response of the tendon. Additionally, it makes it more susceptible to inflammation and injury [6]. Additionally, a pathologically weakened tendon can cause significant pain and disability. In severe cases, can result in tears, which affects the athletes’ performance or even ends the sports career [1]. 

The diagnostic criteria of PT include (but are not limited to) (1): Magnetic reasoning imaging [7]. (2): Functional and pain measurement questionnaire (Victorian Institute of Sport Assessment [VISA]) [7]. (3): Symptomatic measurement questionnaire (Numeric Pain Rating Scale Worst/Usual [NPS-W; NPS-U]) [8]. (4): Pain in the inferior pole of the proximal part of the patellar tendon [4]. (5): Distinct palpation tenderness corresponding to the painful area [9].

The burden of PT on society tends to be underestimated [10]. High economic impacts of PT are usually incurred by the productivity loss of workers and their compensations for seeking medical help [11]. Furthermore, a high prevalence of PT has already been observed in basketball (45%) and volleyball (32%) players due to their exercise modalities (e.g., frequent jumping) [5,12]. It is still challenging to find a way to enable full recovery of individuals with PT. Thus, identifying the risk factors that are easily measured as the prevention tools are of great significance [13].

Nine anthropometric indexes from one systematic review has documented low to very low evidence that BW, BMI, waist-to-hip ratio, leg length difference, the arch height of the foot, quadriceps flexibility and strength, hamstring flexibility, quadriceps strength, and vertical jump performance are the potential risk factors for the development of PT with unrestricted participants’ categories [14]. The findings were justified by identifying and including articles investigating the risk factors associated with PT and comparing individuals with PT with a group of healthy counterparts. However, none of the risk factors identified above have been shown to directly related to PT, which may be due to the lack of consideration for sports modalities of the investigated population. In addition, Fairley et al. (2014) identified an association between obesity and magnetic reasoning imaging diagnosed PT [7]. Fairley et al. (2014) recruited 297 participants aged between 50 and 79 years of age with no history of knee pain from an existing community-based cohort and instructed them to report their weight from 18- to 21 years of age, current BW & BMI, and the heaviest lifetime weight. However, the methods used for data collection lack objectivity [7].

High-quality studies that identify the risk factors for PT, focusing on specific and well-defined population groups is limited [15]. Moreover, evidence has shown that an elevated BMI is associated with musculoskeletal pain due to systematic inflammation, indicating it is worth investigating the relationship between BMI and musculoskeletal pain [16]. Additionally, Stovitz et al. (2008) also demonstrated that BW is a risk factor for musculoskeletal pain in obese children in back and lower extremities [17]. Therefore, this study aimed to systematically review the cross-sectional and prospective cohort studies that investigate the association between BW and BMI and PT in the target population. It is hypothesised that the BW and BMI are associated with PT in elite basketball and volleyball players.

## 2. Methods

### 2.1. The Methods Followed the PRISMA Guidelines *[18]*

Eligibility criteria [19]:

PICO criteria:

Participants: 16–45 years of age professional basketball and volleyball players competing above the regional level, who have developed PT.

Interventions: BW and BMI.

Comparators: 16–45 years of age healthy professional basketball and volleyball players without PT competing above the regional level.

Outcomes: Presence/Development of PT.

Observational study designs, such as cross-sectional and prospective-cohort studies, were considered to be included in the review. The inclusion criteria were; (1): 16–45 years of age professional basketball and volleyball players competing above the regional level. (2): Investigated risk factors were body weight (kg) and body mass index (kg/m^2^). (3): The outcome was the participants’ presence/development of PT. (4): Experiment setting in the sports field, laboratory, or clinical setting. (5): Participants were diagnosed through medical professions with rigorous tools or self-reports as agreeing on diagnostic criteria can allow consistent outcomes reports across studies (i.e., Victoria Institute of Sport Assessment (VISA), ultra-sound imaging, NPS-W, NPS-U, pain in the inferior pole of the proximal part of the patellar tendon). The VISA scale is a comprehensive index of symptom severity in patients with jumper’s knee, and individuals that score less than 80 points are classified as PT [20]. The presence of the hypoechoic lesion of the patellar tendon through ultrasonography indicates the PT [21]. NPS-U and NPS-W are the pain and disability measures used to assess symptom intensity and functional capacity using an 11-point numerical scale. They are reliable and objective in evaluating anterior knee pain [22]. Identifying pain in the patellar tendon during dynamic or static state could also be good criteria for self-report [23].

Regarding the review characteristics, peer-review articles and grey literature (e.g., conference proceedings, doctoral dissertation, pre-prints) in English versions published between 2000 and 2022 were considered to be included in the review. In addition, studies were excluded with unspecified athletes’ competing levels and diagnostic criteria and lack of clear-indicated *p* values. More specifically, studies with risk factors solely measured based on biomechanical, radiographical, ultrasonographical, or individuals’ self-reports were not considered. Additionally, if *p* values used to indicate whether control and intervention groups were statistically different had not been reported, those studies were also excluded.

### 2.2. Data Sources and Search Strategy

Studies were identified by an electronic search of PubMed, EMBASE, CINAHL, Google-Scholar, and the Healthcare-Management-Information-Consortium (HMIC), the National-Technical- Information-Service (NTIS), ClinicalTrial.gov from 2000 to 2022. In addition, citation chasing was conducted to screen and search relevant studies in the reference lists of all included manuscripts. A preliminary feasibility search informed search terms. Examples of search terms in PubMed are ‘patellar tendon’ OR ‘patellar tendon’ OR ‘patellar tendinopathy’ OR ‘patellar tendinopathy’ OR ‘Jumper’s knee’ (Not: ‘patellar apicitis’ OR ‘patellar apicitis’ OR ‘patellar tenosynovitis’ OR ‘patellar tendosynovitis’ OR ‘ACL’ OR ‘Anterior cruciate ligament’ OR ‘Fracture’) and ‘BMI’ OR ‘Body mass index’ OR ‘Body weight’ OR ‘Body composition’. One reviewer (MD) screened the titles and abstracts based on the inclusion criteria, and relevant, complete reports were assessed for eligibility. The reasons for the exclusion during the screening stage were documented. 

### 2.3. Selection and Data Extraction

The review process was conducted by two reviewers (MD and MM). More specifically, the study screening and selection were completed by one reviewer (MD), while both reviewers completed the data extraction and risk of bias assessment. Discrepancies in data extraction and critical appraisal across reviewers were resolved, and a consensus was achieved. Data extraction for each study included: study design, participants’ nationalities, sample size, participants’ characteristics (e.g., age, gender, body weight, body mass index), and diagnostic criteria. Odds ratios (unadjusted or adjusted), 95% confidence intervals, and *p* values were extracted. The studies’ authors were contacted for further clarification if related data was inaccessible.

### 2.4. Quality Appraisal

Two reviewers assessed the studies’ quality using the new version of the Newcastle-Ottawa Scale (NOS) for cross-sectional and prospective cohort studies modified by [18] based on NOS for non-randomised studies in the meta-analysis [24]. The Cochrane Handbook recommends the appraisal tool for Systematic Review of Intervention [25]. Additionally, NOS is essential for the understanding of the non-randomised studies and helping identifying the potential bias [18]. Self-reported outcomes have been assigned one star because the subjective measurement is as crucial as the objective one in the context. This NOS checklist will assess three domains of the papers: selection of the studied group, comparability of the group and control for confounding factors, and outcome. The total maximum score for studies is ten. Studies with scores of 0–4, 5–6, 7–8, and 9–10 were considered unsatisfied, satisfied, good, and very good, respectively [18].

### 2.5. Effect Measures

The two outcomes, BW and BMI, were both continuous variables. The estimated effect (Cohen’s d value) of BW and BMI is determined by calculating the mean difference between the healthy group and the group with PT, dividing by pooled standard deviation. According to the guideline of the thresholds for interpreting effect size, the d value that follows into the interval of 0.2 and 0.5 is classified as a small effect [26]. The reason for using this threshold and *p* values simultaneously is that though *p*-value is the integrated index reflecting effect size, sample size, and test type [27], it only informs whether the values across groups are significantly different from each other. Therefore, the d-value will be extracted to measure how significant the effect of the risk factors is (i.e., the mean difference between groups in standard score form) [28]. Another index for effect size measurement is the odds ratio. It is used to determine the strength of the association between A (particular exposures) and B (outcomes) [29].

### 2.6. Synthesis Methods

Studies that reported BM or BMI were grouped separately for each outcome synthesis. Since all included studies have reported *p*-value, the sample size of the healthy and patellar tendinopathy group, the effect of risk factors (e.g., body and body mass index) could be calculated, so there is no need to conduct any data conversions. A table was created to tabulate the study characteristics, including potential risk factors, the value of risk factors (mean ± standard deviation), diagnostic criteria, sample size, sports competition level, conclusions, secondary findings, and age.

Two authors (M.D. and M.M.) assessed the included papers from a clinical perspective (e.g., diagnosis, variability in population characteristics) and study methodology to determine whether studies could be pooled together for synthesis. There was significant clinical heterogeneity, and therefore studies will be described narratively.

### 2.7. Certainty Assessment

The GRADE system was used for the certainty assessment. The Cochrane Handbook recommends it for Systematic Review of Intervention [25]. Six domains (risks of bias, publication bias, indirectness, inconsistency, imprecision, and effect size) were considered for the quality assessment. The study design determined the initial certainty; the random control trial is rated high, and the observational trial is classified as low certainty. If there is a serious risk of bias in the evidence, the certainty will be downgraded, whereas if there is a large effect size, the certainty will be upgraded. There are four levels of certainty, very low, low, moderate, and high. The high certainty indicates that the authors have confidence that the actual effect is similar to the estimated effect. 

In contrast, the very low certainty suggests a substantial variation between actual and estimated impact. Additionally, the low and moderate certainty evidence refers to the actual effect that might be markedly different and close to the estimated effect, respectively [30]. The imprecision was assessed regarding the pre-specified effect size threshold, and the Cohen power table was specified in the effect measures section [26].

## 3. Results

### 3.1. Search Strategy

**Figure 1:**
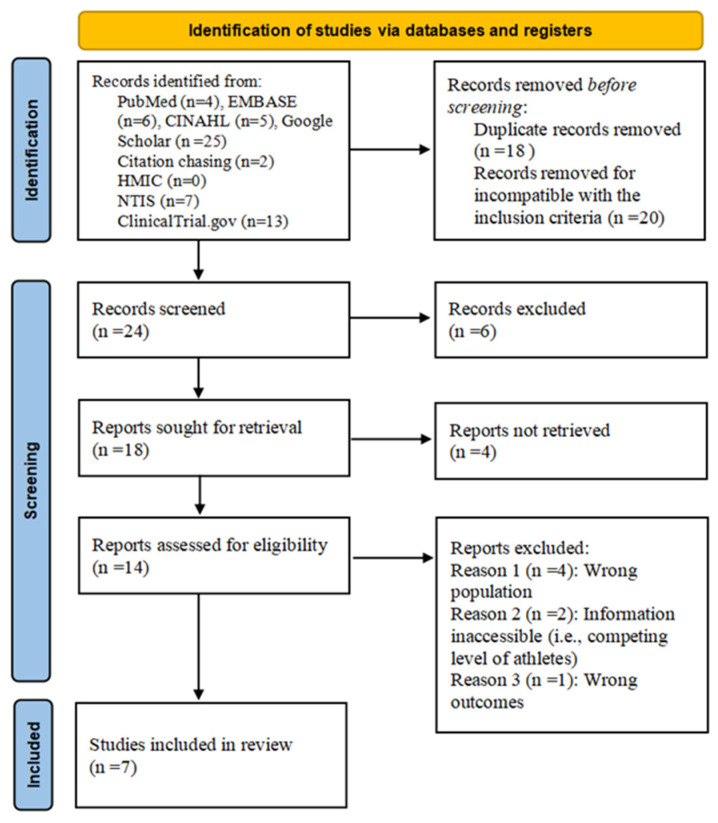
PRISMA flow diagram showing the study selection process [36]. PRISMA, Preferred Reporting Items for Systematic Review and Meta-Analysis.

Figure 1 illustrates the search and retrieval process. After exclusion, 14 studies required full texts to be assessed. Out of these, seven studies were excluded (four studies had the wrong population, two studies’ participants’ characteristics inaccessible, and one study with inappropriate outcomes), resulting in seven studies [8,23,31,32,33,34,35] for inclusion in this review.

The study selection process includes identification, screening, and inclusion. A total of 62 studies [i.e., PubMed (n = 4); EMBASE (n = 6); CINAHL (n = 5); Google Scholar (n = 25); Citation Chasing (n = 2); NTIS (n = 7); HMIC (n = 0); ClinicalTrial.gov (n = 13)] have been identified with 38 being excluded due to duplication (n = 18) and incompatibility with inclusion criteria (n = 20). Ten studies are excluded in the records screening (n = 6) and reports retrieval (n = 4) and 7 studies are excluded in eligibility assessment because of the information inaccessibility (n = 2), wrong population (n = 4) and outcomes (n = 1). Seven studies are included in the review.

### 3.2. Study Characteristics

Table 1 summarises the characteristics and findings of each included study. Two prospective cohort studies and five cross-sectional studies were included. The total number of individuals across the studies was 849 (mean age of 25.5 years), with 436 males and 413 females. Three studies recruited basketball and volleyball players competing at regional or national levels [8,23,31]. Three studies only investigated elite volleyball players [33,34,35]. One study only investigated professional basketball players [32]. The nationalities of the participants were Dutch, Hong Kong, Austria, and Norway. All included studies recruited over 18-year-old participants, except one study mainly focused on teenagers from 16 to 18-year-old [35].

### 3.3. Quality Assessment

Seven included studies were well-design case studies, and cohort studies considered level IV evidence in line with Newcastle-Ottawa Scale (NOS). The results of the quality appraisal are presented in Table 2. According to the guideline, four studies were very good (nine points), two studies were good (eight points), and one study was satisfying (six points). As the non-representative sample and insufficient sample size justification may cause substantial selection bias [8,35], the apparent lack of gender diversity (non-representative samples) across the reports may impair the comparability of the outcomes. Furthermore, a non-representative sample and poor control of the confounding factors were present in one study [32].

### 3.4. Results of Individual Studies and Synthesis

The studies’ results, including investigated potential risk factors, the value of the risk factors, sample size, conclusion (odds ratio and *p*-value with confidence interval), and secondary findings are presented in Table 3. The studies were grouped according to the investigated risk factors (e.g., BM and BMI). Therefore, some studies may be included in both synthesis categories.

### 3.5. Narrative Synthesis

The characteristics of the included studies are presented in Table 1. There was some evidence showing that BW and BMI are risk factors for PT. Five out of seven studies found a negative association between BW and PT (*p* < 0.05) [8,31,32,33,34]. In those studies, the subjects who developed PT tended to be heavier than the healthy group. Three studies reported a non-significant association between BW and PT (*p* > 0.05) [23,34,35]. In addition, three out of four studies found that a higher BMI is associated with the development of PT (*p* < 0.05) [22,23,34]. One study is included in both categories because BW and BMI are the investigated risk factors for PT in the study [34].

A prospective cohort study with 69 males and 72 female elite volleyball players did not observe an association between BW and PT (Healthy group vs. PT group in Mean ± SD: 75.3± 7.8 vs. 76.3 ± 8.5 kg, *p* > 0.05) [35]. However, another cohort study that looked at 142 males and 243 females in elite or non-elite basketball and volleyball with ages between 23 and 30 years (25.3  ±  4.5-year-old in 2008; 28.3  ±  4.5-year-old in 2011) competing at regional or national levels revealed a positive association between BW and the increased presence of PT (as determined by therapists: 63%; Self-reported: 37%) [31]. The reason for the discrepancy in the above two studies may be due to the difference in the competing levels of athletes, as both elite and non-elite athletes with the non-specified number were recruited in the latter trial [31].

A cross-sectional study did not report BW as a determinant of the development of PT [23]. The BW in the PT group (86.7 ± 7.9 kg) is statistically different from the healthy controls (81.9 ± 8.1 kg) (*p* < 0.05) but similar in BMI (Healthy group vs. PT group in Mean ± SD: 22.9 ± 1.9 (PT) vs. 21.8 ± 2.0 kg/m^2^). It remains unclear why those two similar studies had shown different results. This may be attributed to a lack of consideration of the other potential confounding risk factors (e.g., height, training volume, waist-to-hip ratio, muscle tension, flexibility, tibial-length-to-hip ratio, leg strength). In addition, the poor representative of the recruited sample (e.g., gender diversity, sample size, recruiting process) may also explain the inconsistency across the included studies. 

### 3.6. Robustness of the Synthesis Assessment

Due to the qualitative property of the narrative synthesis, the lack of objectivity may affect the robustness of the synthesis. Two independent reviewers were involved in the synthesis process to mitigate the potential bias. Overall, there is very low certainty evidence that a greater BW was positively associated with the increased presence of PT in elite basketball and volleyball players, with an effect size of 0.3, considered to be a small effect, with a 95% confidence interval between 0.9 and 2.1 (Mean ± SD: 72.5 ± 8.4 to 90.1 ± 10.5 kg). There is moderate evidence that increased BMI remained positively associated with the increased incidence of PT in the target population with a 0.25 effect size (small effect) with a 95% confidence interval ranging from 3.7 to 3.2 (Mean ± SD: 21.8 ± 2 to 26.2 ± 3.5 kg/m^2^).

### 3.7. Certainty Assessment

**Figure 2:**
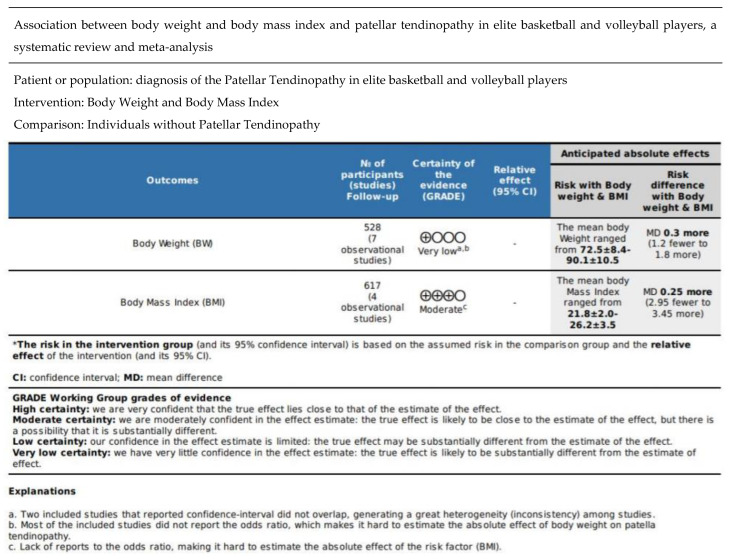
GRADE Summary of Findings Table. a. Two included studies that reported confidence intervals did not overlap, generating a great heterogeneity (inconsistency) among studies. b. Most of the included studies did not report the odds ratio, making it hard to estimate the absolute effect of body weight on patellar tendinopathy. c. Lack of reports on the odds ratio, which makes it hard to estimate the absolute effect of the risk factor (BMI).

The overall evidence certainty for BW and BMI was very low and moderate, respectively. The reasons for the downgrading were listed in the footnotes of the summary of findings, Figure 2.

Very low certainty evidence shows that body weight (BW) is associated with patellar tendinopathy. (i.e., confidence interval of 2 studies do not overlap; lack of odds ratio reporting). A total of 528 participants and 7 observational studies are included for BW. The mean BW ranged from 72.5 ± 8.4 to 90.1 ± 10.5 kg and the mean difference (MD) is 0.3 more (1.2 fewer to 1.8 more). Moderate certainty evidence illustrates that body mass index (BMI) is associated with patellar tendinopathy (i.e., lack of odds ratio reporting). A total of 617 participants and 4 observational studies are included for BMI. The mean BMI ranged from 21.8 ± 2.0 to 26.2 ± 3.5 kg and MD is 0.25 more (2.95 fewer to 3.45 more) [37].

## 4. Discussion

This systematic review assessed the association between BW and BMI and PT in elite basketball and volleyball players. This review has found very low certainty evidence that BW is associated with PT, and moderate certainty evidence has been obtained over the relationship between BMI and PT in the investigated population. However, caution should be taken as these findings are based on a low number of heterogeneous studies. 

BW and BMI are the possible risk factors for PT in the target population; their effect size is relatively small. This may be related to the lower level of adipose tissues of elite athletes than recreational athletes and other populations because of the frequent training and compact gaming arrangement [38]. As a result, elite athletes are associated with lower BW and smaller BMI, exerting less loading on their patellar tendon and lowering the effect of those risk factors [39]. Our findings are consistent with another two comprehensive systematic reviews [14,15] due to similar methodologies used throughout. Van der Worp et al. (2011) found low to very low evidence that risk factors (e.g., BW and BMI) were related to the development of PT by comparing individuals with PT and healthy counterparts [14]. Sprague et al. (2018) found that there was a lack of strong evidence for any potentially modifiable factors (body weight) for PT in athletes by including and reviewing 31 articles (six prospective cohort and 25 cross-sectional studies) [15].

Although it is still unclear what causes PT, several well-established mechanisms have been proposed. The mechanical theory usually refers to the patellar tendon’s incomplete healing after the tendon matrix’s damage in excessive musculoskeletal loading (overuse) [40]. Since the loading is taken up by collagen fibrils, at the higher physiological strain, the collagen fibrils would enter into a state where the chance of micro-damage to the collagen fibrils is expected to be higher. Therefore, repeated or prolonged heavy loading exposure would potentially damage the tendon [41]. To support the accumulation theory in the development of PT (e.g., repetitive heavy loading increases the susceptibility to develop PT), Galloway et al. (2013) proposed that the tendon micro-damage due to improper loading could produce localised fibrils damage that physicians can not diagnose till detectable damage occurs [42]. Therefore, repetitive heavy loading with a higher BW or BMI (high load) can induce a more significant accumulation of micro-trauma [43].

## 5. Limitations and Implications

We acknowledge limitations in our review. First, the limited number of studies included with conflicting findings (articles from 2000–2022) in the review lowers the certainty of the evidence, making it less conclusive. Thus, studies with consistent results need to be grouped in future review. Secondly, the diagnostic criteria varied considerably across studies, including (but not limited to) self-report and ultrasonography. In contrast, others lack objectivity, which potentially affects the findings’ external reliability and internal validity, so it might be better to stratify the included studies according to different diagnostic criteria. Thirdly, most of the included studies failed to adjust to the confounding factors for results, making it unlikely to directly measure the effect of risk factors (BW and BMI) on PT. Further, the number of female participants is insufficient across studies. However, the prevalence of PT is higher in male elite athletes than in females [44], greater gender diversity could enable a better generalisation. 

Regarding the study’s implication, physicians, clinicians, or practitioners are expected to treat the findings cautiously because of the low strength of evidence and consider BW and BMI as part of the assessment package and other well-established risk factors to evaluate one’s propensity to PT. Furthermore, co-morbidities of PT, medications (anti-inflammatories and corticosteroid injection), and length of training sessions should continue to be closely monitored during the clinical assessment.

In the future, more high-quality, prospective cohort studies include more risk factors with reasonable control of each of the confounding factors via splitting the results into subgroups (e.g., male/female; unilateral PT/bilateral PT) with more sophisticated statistical procedures (e.g., regression analysis), from lab to real-life setting in different kinds of the population are indicated. Moreover, how each risk factor, such as gender, training volume, waist-to-hip ratio, BW, and BMI, interact with each other to affect the development of PT also requires further investigation in future research. 

## 6. Conclusions

This literature review found positive associations between BW and BMI and PT in elite basketball and volleyball players. However, this is based on seven heterogeneous studies with very low to moderate certainty evidence. In light of the lack of high quality and substantial evidence, these findings are not suggested as the direct diagnostic criteria for PT as the causal relationship between BM and BMI and PT has not been fully established. Therefore, BW and BMI should be considered parts of a robust, clinically reasoned assessment process when evaluating PT in elite basketball and volleyball players.

## Figures and Tables

**Table 1:** Characteristics of the studies [8,23,31,32,33,34,35].

Study (Study Design)	Potential Risk Factors	Diagnostic Criteria	Sample Size (PT%)	Sports Competition Level	Age	Nationality
de Vries et al. (2015) [31] (Prospective study)	BMI and BW Gender, physical demand	(1): Indicate pain in the inferior pole of patellar tendon (2): Diagnose by physician	381 (13%) Male/female = 142/243	Basketball and volleyball players competing at the elite (regional or national) or non-elite level	25.3 ± 4.5 (2008); 28.3 ± 4.5 (2011)	Dutch
Visnes & Bahr (2013) [35] (Prospective study)	Training volume and body composition	(1): History of pain in patellar tendon (2): Tenderness of palpation corresponding to the painful area	141 (28/141) 69 males 72 females	Volleyball players competing at elite level	16–18	Norway
Lian et al. (2003) (cross-sectional study) [33]	BW, activity volume, capable of jumping	(1): History of pain localize to the lower patellar pole or insertion of the quadriceps tendon (2): Distinct palpation tenderness corresponding to the painful area	47 (24/47) All male	Volleyball players competing at elite level	22.4 ± 2.5 (PT) 22.0 ± 4.0 (healthy)	Norway
Zhang et al. (2017) [23] (Cross-sectional study)	BW, BMI, Passive muscle tension	(1): Pain in the inferior pole of the proximal part of the patellar tendon (2): Pain aggregation during single leg squatting and jumping (3): Pain duration longer than 3 months (4): Maximum intensity of pain in the previous week >3 on the visual analog scale. (5): VISA-P score < 80 points 6) Thickening of proximal part of patellar tendon with area of hypoechoic signal on ultrasound imaging.	66 (36/66) All male	Volleyball and basketball players	21.1 ± 4.4	Hong Kong
Crossley et al. (2007) [8] (Cross-sectional study)	BW, BMI, training volume, thigh flexibility and strength	(1): Functional measure (VISA scale) Symptom measures (NPS-W and NPS-U)	58 (27/58) Female: Male = 19:39	Participants in competitive basketball, netball volleyball or tennis	24 ± 6	Dutch
Gaida et al. (2004) [32] (Cross-sectional study)	BW, height, tibial length to stature ratio (UL vs. control), waist-to-hip ratio (UL vs. control)	Ultrasound examination	39 (15/39) All female	Elite basketball players	Unilateral (20 ± 2) Bilateral&Control (21 ± 3)	Australia
Malliaras et al. (2007) [34] (cross-sectional)	BMI, BW, gender, height, waist girth, hip girth, waist-to-hip ratio	Female and male tendon Normal imaging Abnormal imaging UL Abnormal imaging BL	113 (73 male, 40 female)	Competitive volleyball player	Unknown	Australia

**Table 2:** Detailed summary of critical appraisal of included studies using the adapted-version of Newcastle Ottawa Scale (NOS) for assessing the quality of cross-sectional and prospective cohort studies [8,23,31,32,33,34,35].

Author (Year)	Potential Risk Factors	Diagnostic Criteria	Sample Size	Sports Competition Level	Selection	Comparability	Exposure/ Outcome	Total Stars	Study Quality
Prospective cohort studies									
de Vries et al. (2015) [31]	BMI, BW	(1): Indicate pain in the inferior pole of patella tendon (2): Diagnose by physician	381	Basketball and volleyball players competing at the elite (regional or national) or non-elite level	4	2	3	9	Very good study
Visnes & Bahr (2013) [35]	Training volume and body composition	(1): History of pain in patella tendon (2): Tenderness of palpation corresponding to the painful area	141	Volleyball players competing at elite level	3	2	3	8	Good study
Cross-sectional studies									
Malliaras et al. (2007) [34]	BMI, BW, height, waist girth, hip girth, waist-to-hip ratio	Female and male tendon Normal imaging Abnormal imaging UL Abnormal imaging BL	113	Competitive volleyball player	4	2	3	9	Very good study
Gaida et al. (2004) [32]	BW, height, tibial length to stature ratio (UL vs. control), waist-to-hip ratio (UL vs. control)	Ultrasound examination	39	Elite basketball players	3 (included sample not representative)	0 (poor control of confronting factors)	3	6	Satisfactory study
Zhang et al. (2017) [23]	BMI, BW	(1): Pain in the inferior pole of the proximal part of the patella tendon (2): Pain aggregation during single leg squatting and jumping (3): Pain duration longer than 3 months (4): Maximum intensity of pain in the previous week >3 on the visual analog scale. (5): VISA-P score < 80 points (6): Thickening of proximal part of patellar tendon with area of hypoechoic signal on ultrasound imaging.	66	Volleyball and basketball players	4	2	3	9	Very good study
Crossley et al. (2007) [8]	BW, BMI, arch height during maximal weight bearing, leg length difference	(1): Functional measure (VISA scale) Symptom measures (NPS-W and NPS-U)	58	Participants in competitive basketball, netball volleyball or tennis	3 (the recruited sample does not represent the whole population)	2	3	8	Good study
Lian et al. (2003) [33]	BW, activity volume	(1): History of pain localize to the lower patella pole or insertion of the quadriceps tendon (2): Distinct palpation tenderness corresponding to the painful area	47	Volleyball players competing at elite level	4	2	3	9	Very good study

**Table 3:** Summary of results of individual studies [8,23,31,32,33,34,35].

Study (Study Design)	Potential Risk Factors	Value of Risk Factor (BW; BMI) Mean ± SD Unit: Kg; Kg/m^2^	Sample Size (PT%)	Conclusion	Secondary Findings
Statistical significance of main findings (*p* < 0.05)					
de Vries et al. (2015) [31] (Prospective cohort study) Dutch	BMI and BW Gender, physical demand	BW: 76.1 ± 12.6 BMI: 23.6 ± 3.1	381 (13%) Male/female = 142/243	Weight [OR 1.2 95% (1.0–1.3) *p* < 0.05].	Male gender (*p* < 0.05) [odds ratio (OR) 2.0, 95% confidence interval (CI) 1.1–3.5] Physical demand work (OR 2.3, 95% CI 0.9–6.3)
Lian et al. (2003) (cross-sectional study) [33] Norway	BW, activity volume, capable of jumping	86.7 ± 7.9(PT) 81.9 ± 8.1(healthy)	47 (24/47) All male	Weight is associated with PT (*p* < 0.05)	Weight training (*p* < 0.05) Composite jumping score (*p* < 0.05)
Zhang et al. (2017) [23] (Cross-sectional study) Hong Kong	BW, BMI, Passive muscle tension	BW: 74.1 ± 6.6(PT) 72.5 ± 8.4(control) BMI: 22.9 ± 1.9(PT) 21.8 ± 2.0(control)	66 (36/66) All male	BMI (*p* < 0.05)	Tension of vastus lateralis is associated with PT (r = 0.38; *p* < 0.05)
Crossley et al. (2007) [8] (Cross-sectional study) Dutch	BW, BMI, training volume, thigh flexibility and strength	BW: 80 ± 16 (unilateral PT); 82 ± 14 (bilateral PT) BMI: 25.2 ± 4 26.2 ± 3.5	58 (27/58) F:M = 19:39	Weight (*p* < 0.05) BMI (*p* < 0.05)	Training volume (*p* < 0.05) Thigh flexibility (greater in bilateral PT) *p* < 0.05 Thigh strength (bilateral PT has greater force production) *p* < 0.05
Gaida et al. (2004) [32] (Cross-sectional study) Australia	BW, height, tibial length to stature ratio (UL vs. control), waist-to-hip ratio (UL vs. control)	BW: 74 ± 13	39 (15/39) All female	Weight (*p* < 0.05)	Tibial length to stature ratio was 1.3 above zero in unilateral group. (*p* < 0.05) Waist-to-hip ratio was 0.66 SD above zero in unilateral group. (*p* > 0.05) Leg is weaker in the path
Malliaras et al. (2007) [34] (cross-sectional) Australia	BMI, BW, gender, height, waist girth, hip girth, waist-to-hip ratio	Male: BW:87.2 ± 12. BMI 24.8 ± 2(unilateral) 90.1 ± 10.5. BMI 25.7 ± 2.6(bilateral)	113 (73 male, 40 female)	Male BW and BMI (*p* < 0.05)	Waist-to -hip ratio, waist and hip girth in male (*p* < 0.05)
Statistical significance of main findings (*p* > 0.05)					
Visnes & Bahr (2013) [35] (Prospective cohort study) Norway	Training volume and body composition	BW: 75.3± 7.8(healthy) 76.3 ± 8.5(PT)	141 (28/141) 69 males 72 females	Weight is not associated with PT (*p* > 0.05) OR: 3.2 (−0.9,3.7)	Training volume (increase every hour): (OR) 1.72 (1.18–2.53)
de Vries et al. (2015) [31] (Prospective cohort study) Dutch	BMI and BW Gender, physical demand	BW: 76.1 ± 12.6 BMI: 23.6 ± 3.1	381 (13%) Male/female = 142/243	BMI is not associated with PT. [OR 1.1 (1.0–1.2) (*p* > 0.05)]	Male gender (*p* < 0.05) [odds ratio (OR) 2.0, 95% confidence interval (CI) 1.1–3.5] Physical demand work (OR 2.3, 95% CI 0.9–6.3)
Zhang et al. (2017) [23] (Cross-sectional study) Hong Kong	BW, BMI, Passive muscle tension	BW: 74.1 ± 6.6 (PT) 72.5 ± 8.4 (control) BMI: 22.9 ± 1.9 (PT) 21.8 ± 2.0(control)	66 (36/66) All male	Weight (*p* > 0.05)	Tension of vastus lateralis is associated with PT (r = 0.38; *p* < 0.05)
Malliaras et al. (2007) [34] (cross-sectional) Australia	BMI, BW, gender, height, waist girth, hip girth, waist-to-hip ratio	Male: BW:87.2 ± 12. BMI 24.8 ± 2(unilateral) 90.1 ± 10.5. BMI 25.7 ± 2.6(bilateral)	113 (73 male, 40 female)	Female BW and BMI (*p* > 0.05)	Waist-to-hip ratio, waist and hip girth in male (*p* < 0.05)

## Data Availability

Not applicable.
